# *PLC*-Mediated Signaling Pathway in Pollen Tubes Regulates the Gametophytic Self-incompatibility of *Pyrus* Species

**DOI:** 10.3389/fpls.2017.01164

**Published:** 2017-07-06

**Authors:** Haiyong Qu, Yaqin Guan, Yongzhang Wang, Shaolin Zhang

**Affiliations:** ^1^College of Horticulture, Qingdao Agricultural UniversityQingdao, China; ^2^College of Horticulture, Nanjing Agricultural UniversityNanjing, China

**Keywords:** phospholipase C, S-RNase, gametophytic self-incompatibility, IP_3_, pollen tube

## Abstract

Among the Rosaceae species, the gametophytic self-incompatibility (GSI) is controlled by a single multi-allelic S locus, which is composed of the pistil-S and pollen-S genes. The pistil-S gene encodes a polymorphic ribonuclease (S-RNase), which is essential for identifying self-pollen. However, the S-RNase system has not been fully characterized. In this study, the self-S-RNase inhibited the Ca^2+^-permeable channel activity at pollen tube apices and the selectively decreased phospholipase C (*PLC*) activity in the plasma membrane of *Pyrus pyrifolia* pollen tubes. Self-S-RNase decreased the Ca^2+^ influx through a *PLC*-mediated signaling pathway. Phosphatidylinositol-specific *PLC* has a 26-amino acid insertion in pollen tubes of the ‘Jinzhuili’ cultivar, which is a spontaneous self-compatible mutant of the ‘Yali’ cultivar. ‘Yali’ plants exhibit a typical S-RNase-based GSI. Upon self-pollination, *PLC* gene expression is significantly higher in ‘Jinzhuili’ pollen tubes than that in ‘Yali’ pollen tubes. Moreover, the *PLC* in pollen tubes can only interact with one of the two types of S-RNase from the style. In the *Pyrus x bretschneideri* Rehd, the *PLC* directly interacted with the S_7_-RNase in the pollen tube, but not with the S_34_-RNase. Collectively, our results reveal that the effects of S-RNase on *PLC* activity are required for S-specific pollen rejection, and that *PLC*-IP_3_ participates in the self-incompatibility reaction of *Pyrus* species.

## Introduction

Self-incompatibility (SI) is an important mechanism that prevents self-fertilization and maintains genetic diversity in flowering plants (Silva and Goring, 2001). Gametophytic self-incompatibility (GSI) is the most common SI system (Franklin-Tong and Franklin, 2003), which has been described in >60 families of flowering plants (Kao and McCubbin, 1996). Only two GSI systems have been elucidated in detail at the molecular level (Franklin-Tong and Franklin, 2003; De Franceschi et al., 2012). One GSI mechanism, so far found only in the *Papaveraceae*, has a small secreted peptide–the S-protein (PrsS: *Papaver rhoeas* style S) as its pistil S-component. PrpS (*P. rhoeas* pollen S) is the *Papaver* pollen S determinant (Wheeler et al., 2009), but the function of PrpS is unclear yet (Fujii et al., 2016). Pollen growth can be inhibited within minutes by placing it on the stigma. An important finding is that the S-protein system includes a Ca^2+^-dependent signaling network. Self-incompatibility triggers an influx of extracellular Ca^2+^ into incompatible pollen tubes (Franklin-Tong et al., 2002), and the apical Ca^2+^ gradient rapidly disappears with the cessation of pollen tube growth during GSI (Franklin-Tong et al., 1997). The other GSI mechanism, which has been detected in the Solanaceae, Rosaceae and Scrophulariaceae families, has S-RNase as the pistil S-component and an F-box protein (SLF) as the pollen S-component (Zhang and Hiratsuka, 1999; Yang et al., 2007). In all species exhibiting GSI, incompatible pollen tubes will not grow beyond one-third of the length of the style. The self-S-RNase enters pollen tubes with the assistance of ABC transporters in apple (Meng et al., 2014) and self-S-RNase degrades ribosomal RNA (Quinet et al., 2014). However, RNA degradation is not the only SI event. S-RNase specifically induces tip-localized reactive oxygen species disruption, actin cytoskeleton depolymerisation, and nuclear DNA degradation in incompatible pear pollen tubes (Liu et al., 2007; Wang et al., 2010). Nevertheless, it is unclear whether S-RNase triggers alterations of pollen tube elements to inhibit incompatible pollen tube growth in a direct or indirect way. It has been suggested that pollen rejection systems and incompatibility systems are interconnected, and should be regarded as different parts of a single system that provides fine control over plant fertilization (Cruz-Garcia et al., 2003). Free cytosolic Ca^2+^ ([Ca^2+^]_i_) in pollen tubes is a well-established second messenger responsible for transducing directional growth signals (Malhó et al., 1994; Steinhorst and Kudla, 2013). A tip-focused apical [Ca^2+^]_i_ gradient is a feature of growing pollen tubes (Pierson et al., 1996). Moreover, IP_3_ (inositol 1, 4, 5-triphosphate)-mediated [Ca^2+^]_i_ signals are precisely controlled in both time and space, resulting in a signaling system that is simultaneously ubiquitous, versatile, and specific (Mak et al., 2000). Inositol 1, 4, 5-trisphosphate and other inositol lipid-related compounds have been proposed as important regulators of pollen tube growth (Zonia and Munnik, 2004; Monteiro et al., 2005). In addition, phosphatidylinositol-specific phospholipase C (*PI-PLC*) regulates tip growth by controlling IP_3_-gated Ca^2+^ fluxes and altering the spectrum of PI lipids in the tube apex (Dowd et al., 2006; Heilmann and Ischebeck, 2016). Thus, there are close links between *PLC*, IP_3_ production, and Ca^2+^ gradient during pollen tube growth.

Self- and non-self-recognitions are vital to the survival of all living organisms ranging from bacteria to humans. There are many biological events in the process of recognition, including immune defense and mate choice. Self- and non-self-discriminations are involved in mate choice (Li et al., 2017). Pollen–stigma interactions are similar to host–pathogen interactions (Hodgkin et al., 1988; Kovaleva et al., 2002; Kovaleva and Zakharova, 2003; Wu et al., 2008; Wilkins et al., 2014). When plants are under the stress of biotic and abiotic factors, the activity of *PLC* is rapidly activated, and the expression of *PLC* gene was up-regulated (Vossen et al., 2010).

In the Maloideae of Rosaceae, S-locus F-box genes appear to be involved in the determination of pollen S specificity. However, despite the fact that several models have been developed to explain how the two components of the S-locus interact, the mechanism by which SLF/SFB (*S-haplotype-specific F-box*) and S-RNase interact to trigger the SI reaction remains uncertain (Akagi et al., 2016; Ashkani and Rees, 2016). Moreover, the speculative GSI models based on SLF are often paradoxical. More and more data suggest that other loci have crucial influence on the sexual (in) compatible phenotype (Hegedűs et al., 2012). As has been proven by the finding of unexpected diversity in molecular mechanisms adopted for SI, further studies are likely to uncover novel forms of molecular interactions (Fujii et al., 2016). The purpose of this study is to explore the effect of S-RNase on Ca^2+^ channels, which has been identified in our published paper (Qu et al., 2007) on phospholipase C activity and its gene expression in the apical pollen tube. In doing so, we assessed *PLC* function in GSI, and developed a new model to explain our results.

## Results

### S-RNase Effects on Ca^2+^ Gradients and Currents

**FIGURE 1:**
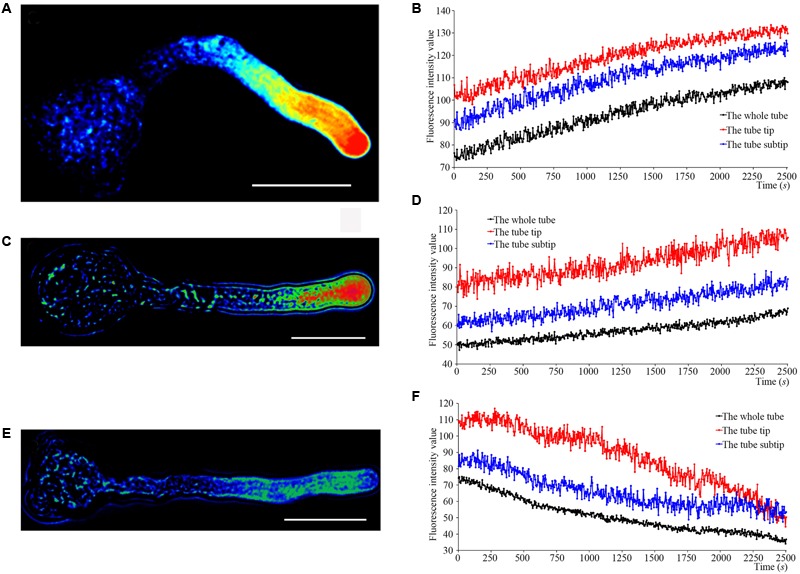
S-RNase affects the Ca^2+^ gradient at the pollen tube tip of ‘Housui.’ After adding S-RNase to the pollen tube culture medium, we observed the changes of Ca^2+^ gradient at apical pollen tube. **(A,B)** Variation in [Ca^2+^]_c_ in an untreated pollen tube (control). The image is of a pollen tube stained with fluo-4/AM. **(C,D)** Variation in [Ca^2+^]_c_ in a pollen tube treated with 1.0 μg μl^-1^ non-self-S-RNase (‘Imamuraaki’). **(E,F)** Variation in [Ca^2+^]_c_ in a pollen tube treated with 1.0 μg μl^-1^ self-S-RNase (‘Housui’). Scar bar is 60 μm in **(A,C,E)**.

When fluo-4/AM was loaded into ‘Housui’ pollen tubes at room temperature, the normally growing pollen tubes preserved the Ca^2+^ gradient at the tube apex (**Figures 1A,B**). It had no effect on the apical Ca^2+^ gradient by adding 1.0 μg μl^-1^ non-self-S-RNase (‘Imamuraaki’) (**Figures 1C,D**). However, after adding 1.0 μg μl^-1^ self-S-RNase (‘Housui’), it decreased [Ca^2+^]_i_ at the pollen tube tips and sub-tips (**Figures 1E,F**). These results indicate that self-S-RNase can decrease Ca^2+^ concentrations in pollen tube apices and disrupt Ca^2+^ gradients.

**FIGURE 2:**
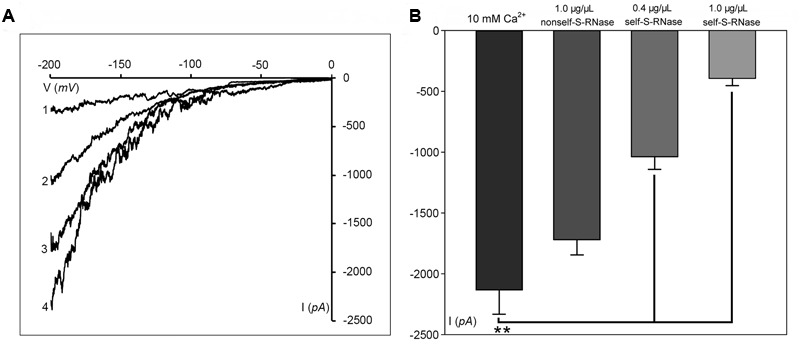
S-RNase affects Ca^2+^ currents in the pollen tube tip plasma membrane. **(A)** The representative recording currents trace from ramp voltage clamping. Different treatment with (1) 1.0 μg μl^-1^ self-S-RNase (‘Housui’), (2) 0.4 μg μl^-1^ self-S-RNase, or (3) 1.0 μg μl^-1^ non-self-S-RNase (‘Imamuraaki’), (4) 10 mM Ca^2+^ in bath solutions. **(B)** Summary of experiments in **(A)**. Comparing the current at the standard of –200 mV. Comparison between self-S-RNase (0.4 and 1.0 μg μl^-1^, both *n* = 14) with non-self-S-RNase (1.0 μg μl^-1^, *n* = 14). The data are presented as the mean ± standard error. ^∗∗^*p* < 0.01.

We determined Ca^2+^ currents in ‘Housui’ pollen tube apex protoplasts with whole-cell recordings 30 min after adding 1.0 μg μl^-1^ (final concentration) ‘Imamuraaki’ non-self-S-RNase into the bath solution, and replacement with 0.4 or 1.0 μg μl^-1^ ‘Housui’ self-S-RNase. Compared to controls with 10 mM Ca^2+^, non-self-S-RNase had no effect on Ca^2+^ currents. However, in the presence of ‘Housui’ self-S-RNase, the calcium ion current presented very significant decrease (**Figure 2A**). The current amplitudes of 1.0 μg μl^-1^ non-self-S-RNase were >4-fold bigger than the amplitudes of 1.0 μg μl^-1^ self-S-RNase at a reference voltage of -200 mV (**Figure 2B**). These results suggest that self-S-RNase was selective in its regulation of Ca^2+^ channel activity in pollen tube protoplast membranes.

### IP_3_ Regulation of Ca^2+^ Channel Activity in Pollen Tubes

**FIGURE 3:**
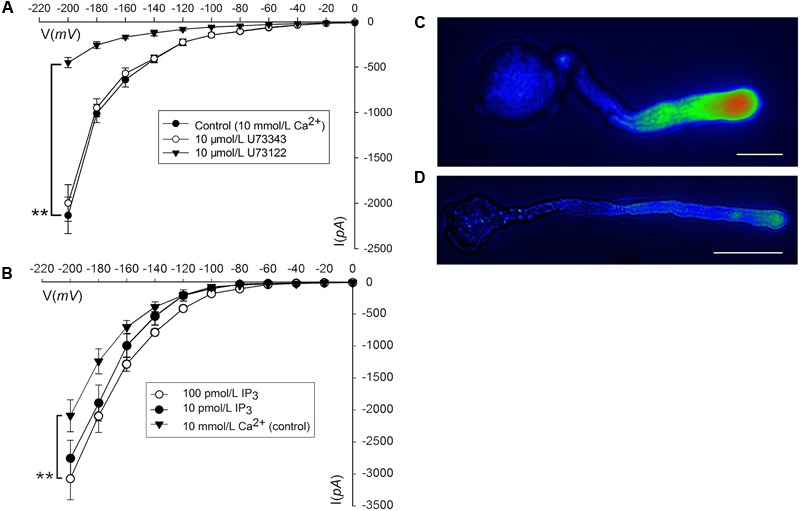
IP_3_-regulated hyperpolarization-activated Ca^2+^ channels and tip-focused Ca^2+^ gradient at the pollen tube apex. Ca^2+^ currents in ‘Housui’ pollen tube protoplasts were recorded by using a step pattern with basal pipette solutions of U-73122, U-73343, or IP_3_ at various concentrations. The image of pollen tube was stained with fluo-4/AM. **(A)** Ca^2+^ currents were compared among basal pipette solutions with and without U73343 or U73122 based on I/V. Data of pollen tube apex protoplasts were recorded for each concentration and the means ± standard errors were calculated. We also compared the current at –200 mV. U-73122 (10 μM, *n* = 11), U-73343 (10 μM, *n* = 9), and Control (10 mM Ca^2+^, *n* = 15). ^∗∗^*p* < 0.01. **(B)** Effects of IP_3_ in pipettes were recorded by using ramp voltage clamping, and they were compared with the effects on Control (10 mM Ca^2+^, *n* = 15). We also compared the current at –200 mV with pipette solutions containing IP_3_ at two different concentrations (100 and 10 pM, *n* = 18 and 14) respectively. ^∗∗^*p* < 0.01. **(C)** Image of the tip-focused Ca^2+^ gradient in a pollen tube exhibiting normal growth. **(D)** The Ca^2+^ gradient was eliminated when 10 μM (final concentration) U-73122 was added to the culture medium. Scar bar is 35 μm in **(C)** and 50 μm in **(D)**.

Phosphatidylinositol-specific phospholipase C controls the levels of PIP_2_ (i.e., substrate; phosphatidylinositol 4,5-bisphosphate) and IP_3_ (i.e., cleavage product), both of which are strong tip growth regulator candidates (Helling et al., 2006). We recorded the Ca^2+^ currents in pollen tube apices with our established method (Qu et al., 2007). When U-73122, an inhibitor of *PLC* activity (Mogami et al., 1997; Evdonin et al., 2004), was added to protoplasts through a pipette at a final concentration of 10 μM, the Ca^2+^ currents significantly decreased (*p* < 0.01). The current amplitudes, which were assessed at a standard reference voltage of -200 mV, were approximately sixfold smaller than those of the controls (**Figure 3A**). After adding U-73343-a biologically inactive analog of U-73122, it did not show significant difference from the control (*p* > 0.01) (**Figure 3A**). We observed that compared to controls, IP_3_ increased Ca^2+^ currents when the final concentration was 10 or 100 pM in the pipette solution, with a greater effect at 100 pM (**Figure 3B**). Moreover, U-73122, with a final concentration of 10 μM in culture medium, also disrupted tip-focused Ca^2+^ gradients of pollen tubes, as revealed by fluo-4/AM staining (**Figures 3C,D**). These results suggest that hyperpolarization-activated Ca^2+^ channels at the tips of pollen tubes were regulated by IP_3_, and that *PLC* regulated the tip-focused Ca^2+^ gradient of pollen tubes.

### S-RNase-Specific Decreases in *PLC* Activity

**FIGURE 4:**
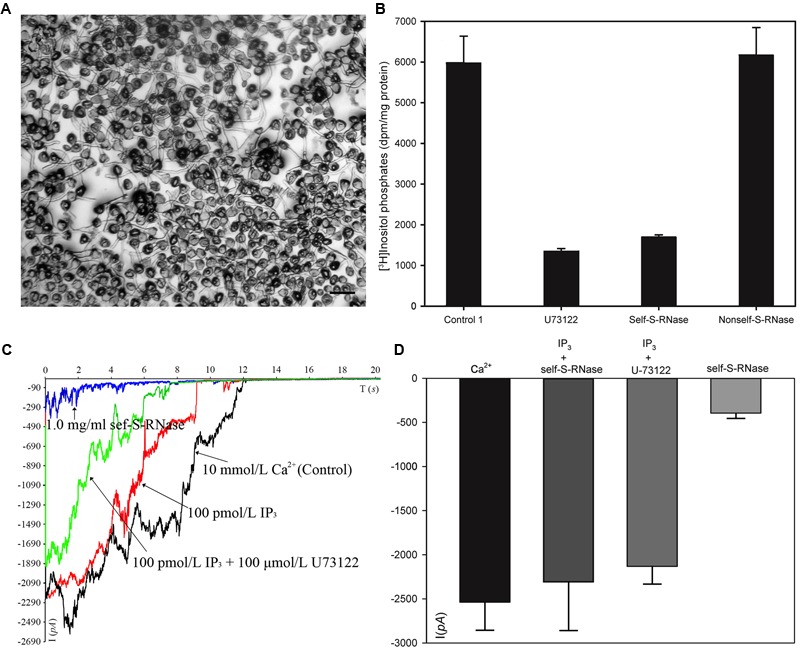
S-RNase affected *PLC* activity in pollen tubes and IP_3_-regulated Ca^2+^ channels of pollen tube apices. **(A)** Membrane was extracted from pollen tubes (‘Housui’) grown for 3 h with a germination rate of >80%. The scale bar corresponds to 200 μm. **(B)** [^3^H]-IP_3_ was measured after membrane protein cleavage to [^3^H] PI 4,5-P_2_PIP2 (control). The presented data are the averages of three or four independent experiments, and are presented as the mean ± standard error. **(C)** Ca^2+^ currents were recorded in response to a hyperpolarizing voltage ramp from 0 to –200 mV (ramp speed 9.84 mV s^-1^). The black curve represents the current when the extracellular solution contained 10 mmol/L Ca^2+^ (control). The red curve refers to the current when the pipette solutions contained 100 pmol/L IP_3_ and bath solution contained 1.0 mg ml^-1^ self-S-RNase (‘Housui’) at the same time. The green curve represents the current when the pipette solutions contained a mixture of 100 pmol/L IP_3_ and 10 μmol/L U-73122. The blue curve represents the current when the extracellular solution contained 1.0 mg ml^-1^ self-S-RNase. **(D)** The currents were compared at –200 mV for the three treatments.

We collected plasma membrane fractions (containing cell-membrane Phospholipase C) from ‘Housui’ pollen tubes (**Figure 4A**), and analyzed the influence of ‘Housui’ self-S-RNase and ‘Imamuraaki’ non-self-S-RNase on its activity. *PLC* activity was monitored *in vitro* by incubating plasma membrane fractions with ^3^H-PIP_2_ and measuring the labeled product in the soluble fraction (presumably IP_3_) (**Figure 4B**). It remarkably decreased *PLC* activity by adding 10 μM (final concentration) U-73122. However, 1 μg μl^-1^ (final concentration) self-S-RNase also severely inhibited *PLC* activity, while non-self-S-RNase of the same final concentration only had minimal effect on the *PLC* activity (**Figure 4B**). These results suggest that self-S-RNase inhibited *PLC* activity in self-pollen tubes.

When the basal pipette solution contained 100 pM IP_3_, Ca^2+^ currents in ‘Housui’ protoplasts (whole-cell configuration) were fully recovered from the suppression caused by the presence of 1 μg μl^-1^ ‘Housui’ self-S-RNase in the bath solution. Furthermore, compared to the control, no significant differences were found in current when the pipette solution contained a mixture of 10 μM U-73122 and 100 pM IP_3_. These results indicate that IP_3_ enabled the recovery of Ca^2+^ currents previously suppressed by U-73122 (**Figures 4C,D**). The data suggest that self-S-RNase suppressed the activity of Ca^2+^ channels at pollen tube tips by inhibiting *PLC* activity and decreasing IP_3_ levels in protoplasts.

### *PCL* Interacts with S-RNase

**FIGURE 5:**
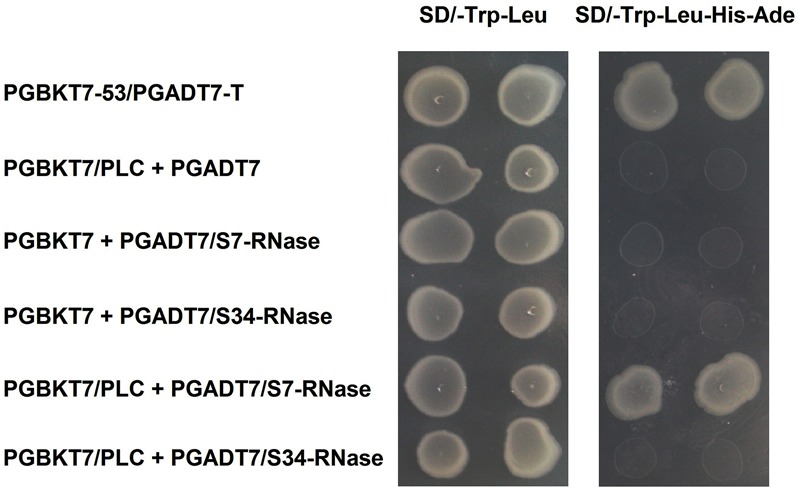
Interaction of *PLC* in the pollen tube with S-RNase in the style of *Pyrus x bretschneideri* Rehd in yeast two-hybrid assays. Positive control (PGBKT7-53/PGADT7-T); Negative control (PGBKT7/PLC + PGADT7; PGBKT7 + PGADT7/S7-RNase; PGBKT7 + PGADT7/S34-RNase); Experimental group (PGBKT7/PLC + PGADT7/S7-RNase; PGBKT7/PLC + PGADT7/S34-RNase). The SD/-Trp/-Leu/X-α-Gal (DDO/X, **Right**) and SD/-Trp/-Leu/-His/-Ade/X-α-Gal (QDO/X, **Left**) plates were incubated at 30°C for 3 days and then visualized. Co-transformed yeast containing pGBKT7/PLC and pGADT7/S_34_-RNase plasmids grew on the SD medium without Trp and Leu, but it could not grow on SD medium lacking Trp, Leu, His, and Ade. Yeast cells with pGBKT7/PLC and pGADT7/S_7_-RNase grew on SD medium lacking additional nutrients.

**FIGURE 6:**
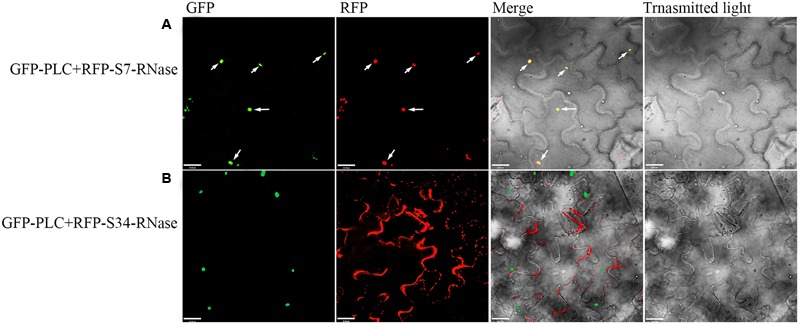
Colocalization of the PLC with S-RNase. Dual-view analyses of cells expressing both GFP-PLC (removing the C2 region bound to the membrane) and RFP-S-RNase. **(A)** Co-transformed with GFP-PLC and RFP-S7-RNase. **(B)** Co-transformed with GFP-PLC and RFP-S34-RNase. The images of GFP fluorescence, DesRed fluorescence, brightfield, and the merged were obtained through fluorescence microscopy, and were displayed from left to right respectively. The merged image (yellow) shows the high degree of colocalization.

‘Dangshansuli’ (*Pyrus x bretschneideri* Rehd.) is the most important commercial Asian pear cultivar, with a production volume of 4 million tons per year. The draft genome of the ‘Dangshansuli’ was the first comprehensive fine pear genome (Wu et al., 2012b). Its S-genotype is *S_7_S_34_.* To further identify possible *PLC* and S-RNase interactions, we performed yeast two-hybrid experiments. The *PLC* coding region was subcloned into the pGBKT7 vector, and it was tested against *S_7_-RNase* and *S_34_-RNase* pGADT7 fusion constructs. Full-length *PLC* interacted with full-length *S_7_-RNase*, but not with *S_34_-RNase* (**Figure 5** and Supplementary Figure S1). To further gain insights into the relationship between PLC and S-RNase, we transiently expressed GFP-PLC and RFP-S-RNase in tobacco leaves. Based on the fluorescence microscopy, we found that when fused to GFP, the same PLC fragment used in the yeast-two-hybrid experiment (without the membrane-associated C2 region) mainly localized near the nucleus, and the signal overlapped with the DsRed fluorescence emitted by the RFP-S7-RNase (**Figure 6A**). However, it did not result in fluorescence superposition by expressing RFP-S34-RNase and GFP-PLC in tobacco cells (**Figure 6B**). These results suggest that *PLC* interacted with the products of multiple alleles of one S-RNase, rather than the products of two S-RNases.

### *PLC* Polymorphisms in Pollen Tubes

**FIGURE 7:**
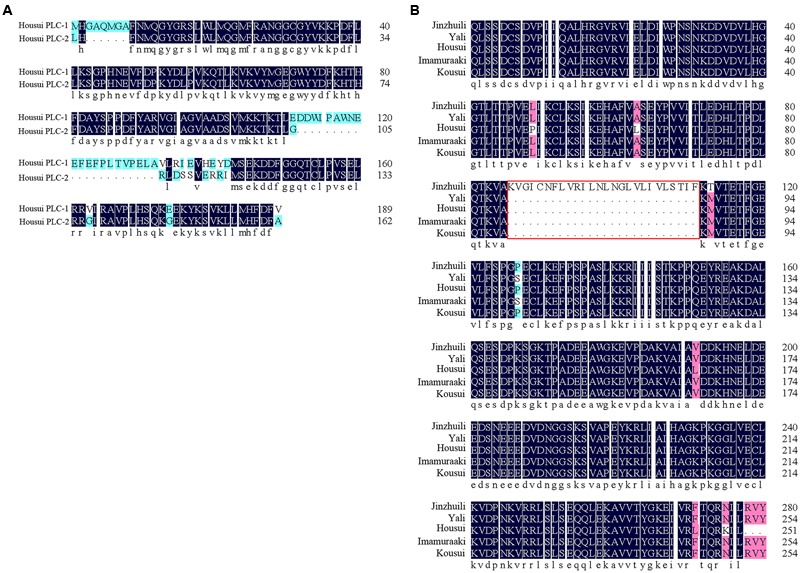
Partial *PLC* amino acid sequence alignment. **(A)** Amino acid sequence of the ‘Housui’ partial Y-domain and subsequent amino acids. **(B)** Partial C2- and Y-domains of *PLC*. The amino acid sequence of self-compatible ‘Jinzhuili’ was compared with that of SI varieties (‘Housui,’ GenBank: EU295486.1).

Self-specific direct interactions between *PLC* and self-S-RNase require *PLC* polymorphisms at a level matching the S-RNase polymorphisms. To clone the *PLC* cDNA from *Pyrus pyrifolia* pollen tubes, one degenerate oligonucleotide, designed according to a conserved amino acid sequence, was used for PCR analyses with cDNA from ‘Housui’ pollen tubes as templates. Two DNA fragments (approximately 750 bp) were amplified. A BLAST analysis indicated that these two fragments were homologous to plant *PI-PLCs*. Sequence alignments of the two DNA fragments revealed the encoded amino acid sequences (according to Open Reading Frame Finder^1^) with a polymorphism frequency of 22% (**Figure 7A**). The two partial *PLC* genes were determined to be *PI-PLC 2-like* (*LOC103936790*) according to Protein Blast (*P. x bretschneideri*), with identities of 96 and 83%.

For fruit trees, including pear trees, it is very difficult to construct a stable transgenic system. However, we obtained ‘Jinzhuili’ as a naturally occurring self-compatible mutant of ‘Yali.’ Field pollination tests showed that >76% of flowers set fruits, and many pollen tubes grew down to the base of the style during ‘Jinzhuili’ self-pollination and cross-pollination of ‘Yali’ (female) × ‘Jinzhuili’ (male). In contrast, during ‘Yali’ self-pollination and cross-pollination of ‘Jinzhuili’ (female) × ‘Yali’ (male), fruit set did not occur, and most pollen tubes were arrested at the upper part of the style. The results indicate that ‘Yali’ and ‘Jinzhuili’ had a normal SI response in the style, which rejected competent ‘Yali’ pollen, but accepted ‘Jinzhuili’ pollen. Further molecular analyses revealed that both ‘Yali’ – and ‘Jinzhuili’ – labeled *S_21_-RNase* and *S_34_-RNase* genes were transcribed normally with identical mRNA sequences. Therefore, we suggest that it was most likely ‘Jinzhuili’ had lost pollen SI activity, resulting in a style that behaved normally during the SI response, similar to that of the wild-type ‘Yali’ (Supplementary Figure S2).

**FIGURE 8:**
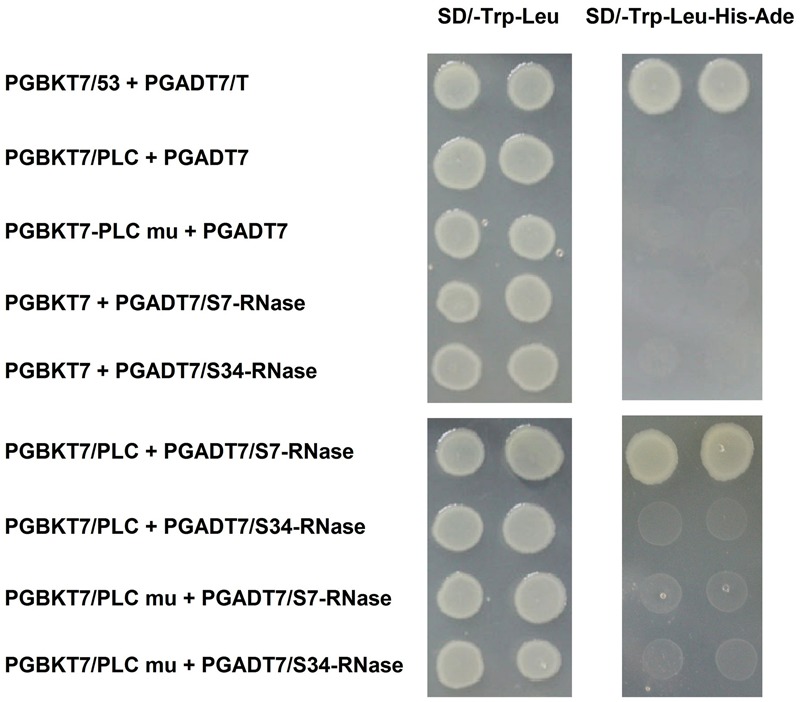
Interaction of inserting the mutated *PLC* (*PLC* mu) into the pollen tube with S-RNase in the style of *P. x bretschneideri* Rehd in yeast two-hybrid assays. Positive control (PGBKT7-53/PGADT7-T); Negative control (PGBKT7/PLC + PGADT7; PGBKT7/PLC mu + PGADT7; PGBKT7 + PGADT7/S7-RNase; PGBKT7 + PGADT7/S34-RNase); Experimental group (PGBKT7/PLC + PGADT7/S7-RNase; PGBKT7/PLC + PGADT7/S34-RNase; PGBKT7/PLC mu + PGADT7/S7-RNase; PGBKT7/PLC mu + PGADT7/S34-RNase). The SD/-Trp/-Leu/X-α-Gal (DDO/X, **Left**) and SD/-Trp/-Leu/-His/-Ade/X-α-Gal (QDO/X, **Right**) plates were incubated at 30°C for 3 days, and then visualized. Co-transformed yeast containing pGBKT7/PLC and pGADT7/S34-RNase plasmids grew on the SD medium without Trp and Leu, but it could not grow on SD medium lacking Trp, Leu, His, and Ade. Yeast cells with pGBKT7/PLC and pGADT7/S7-RNase grew on SD medium without Trp and Leu, also can grow without Trp, Leu, His, and Ade. The results indicate that there is interaction between *PLC* and S7-RNase proteins, but there is no interaction between *PLC* mu and S7-RNase protein.

We used a previously described method to clone *PLC* genes from ‘Jinzhuili’ and ‘Yali.’ The amino acid sequence alignments (**Figure 7B**) showed that the *PLC* of ‘Jinzhuili’ had a 26-amino acid insertion. We inserted this nucleotide fragment to encode the 26-amino acid peptide segment into the PLC gene of ‘Dangshansuli’ (initially used in the yeast-two-hybrid experiment as shown in **Figure 5**). This mutated PLC protein did not interact with either the S7-RNAse or the S34-RNAse in our yeast-two-hybrid experiment (**Figure 8** and Supplementary Figure S1). The self-S-RNase could not identify mutant *PLC*, and was unable to inhibit *PLC* activity. Therefore, ‘Jinzhuili’ exhibited self-compatibility. These results suggest that the *PLC* in the pollen tube is one of the male determinants in the self-incompatibility reaction.

### Analysis of Gene Expression and Proteome Difference in *PLC* Gene in Pollen Tube

We explored the difference in gene expression at the early stage after pollination, and compared gene expression levels in the styles of ‘Yali’ and ‘Jinzhuili’ 0.5 h after self-pollination. Although the differences in gene expression were compared after pollination, the GO terms associated with “pollen tube growth,” “cell tip growth,” “pollen tube development,” and “pollination” identified cell activities consistent with pollen growth activity (data unpublished). We searched for *Pyrus PLC* genes in the GenBank database (Supplementary Table S1). The differences in gene expression levels were determined based on high-throughput sequencing results (Supplementary Table S1). Importantly, 0.5 h after pollination, the pollen grain had just germinated on the stigma, and there was no difference in pollen tube growth between plants that underwent compatible and incompatible pollinations (Supplementary Figure S3). Therefore, at this time point, differences in gene expression between stigmas derived from compatible and incompatible pollinations likely reflect differences related to the compatibility of the interaction rather than differences in pollen tube size or viability.

**FIGURE 9:**
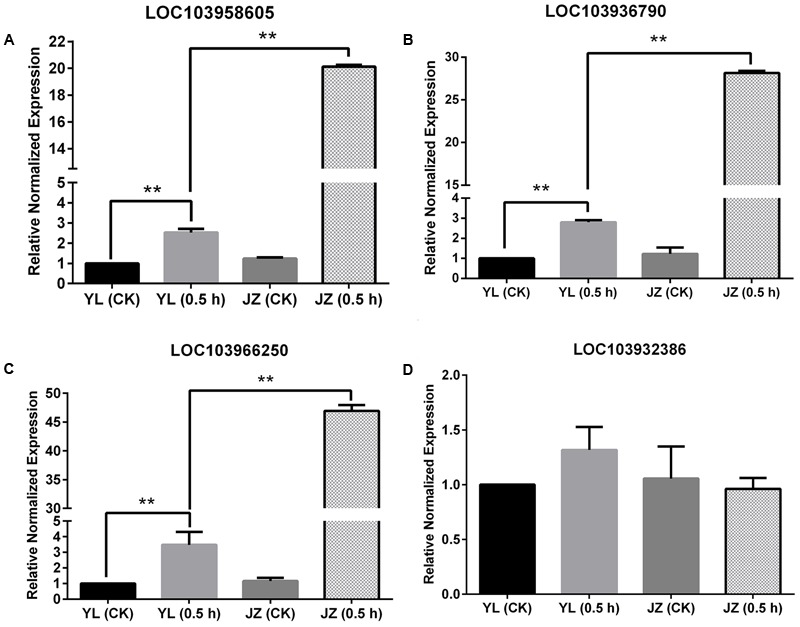
Expression of four *PI-PLC* genes in styles based on quantitative PCR. Comparison of the expression of four *PLC* genes during compatible and incompatible pollination. ‘Yali’ styles were collected 0.5 h after self-pollination [YL (0.5 h)]. ‘Jinzhuili’ styles were collected 0.5 h after self-pollination [JZ (0.5 h)]. Non-pollinated ‘Jinzhuili’ [JZ (CK)] and ‘Yali’ [YL (CK)] styles were collected 0.5 h without pollination. The GeneID of the four *PLC* genes are as follows: **(A)** LOC103958605. **(B)** LOC103936790. **(C)** LOC103966250. **(D)** LOC103932386. ^∗∗^*p* < 0.01. Primer sequences are provided in electronic Supplementary Table S2.

In theory, if the *PLC* gene is expressed specifically in pollen tubes, its expression during compatible pollination should be higher than that of the control or during incompatible pollination. This is because pollen tubes can grow during compatible pollination. The values of Log_2_(Jinzhuili _0.5__h_/ Jinzhuili _CK_) and Log_2_(Jinzhuili _0.5__h_/ Yali _0.5__h_) were positive for only *LOC103958605, LOC103936790*, and *LOC103966250*, although the *p-*value for *LOC103966250* was higher than 0.05 (i.e., 0.252186). Therefore, we selected these three genes for quantitative PCR analysis, and detected the expression levels at 0.5 h after pollination. The expression levels in the pollen tube after compatible pollination were significantly higher (*p* < 0.01) than those after incompatible pollination and the expression level of non-pollinated styles. During compatible pollination, the expression levels were 7, 10, and 14 times higher than those during incompatible pollination (**Figures 9A–C**). Moreover, based on Supplementary Table S1, we selected *LOC103932386*, which exhibited no differential expression, for further RT-PCR analysis. We confirmed that the expression level did not change following pollination (**Figure 9D**). These results suggest that the three genes of *LOC103958605, LOC103936790*, and *LOC103966250*, were expressed specifically in the pollen tube, and they were related to pollen tube growth in the style. The expression levels of these three genes in the styles were higher (*p* < 0.01) after incompatible pollination than in non-pollinated styles.

By using the iTRAQ technique, we measured the style proteome of the ‘Jinzhuili’ and ‘Yali’ after 0.5 h of self-pollination respectively. The expression of *PI-PLC* related proteins was different under ‘Jinzhuili’ (0.5 h) – vs. – ‘Yali’ (0.5 h), ‘Yali’ (0.5 h) – vs. – ‘Jinzhuili’ (CK), ‘Jinzhuili’ (0.5 h) – vs. – ‘Jinzhuili’ (CK) (**Table 1**). In the case of self-compatibility comparing to self-incompatibility, *PI-PLC*-related protein up-regulated expression was significant (Protein ID: XP_009373787.1, XP_009343292.1, XP_009344939.1). The proteins, XP_009343292.1 and XP_009344939.1, were up-regulated only in the case of self-compatibility. In particular, the changes in XP-009344939.1 protein expression were consistent with the results of high-throughput sequencing and RT-PCR. Although expression of the XP-009346269.1 protein was down-regulated in styles derived from compatible pollination, and up-regulated in styles derived from incompatible pollination, the differences were not significant. These results indicate that by using the ITRAQ technology to analyze *PLC* protein expression differences, it could be useful to distinguish between compatibility and incompatibility.

**Table 1:** Analysis of PLC protein expression after pollination.

Comparison	Expression	Protein_ID	Description	Protein coverage	The number of peptides	Log2 (Ratio)	Q-value
Z _0.5 h_/Y_0.5 h_	Up-regulated	XP_009373787.1	PREDICTED: PI-PLC X domain-containing protein At5g67130 [*Pyrus x bretschneideri*]	0.161	5	1.945	0.002
Z _0.5 h_/Y _0.5 h_	Up-regulated	XP_009343292.1	PREDICTED: PI-PLC X domain-containing protein At5g67130-like [*Pyrus x bretschneideri*]	0.174	4	1.523	0.028
Z _0.5 h_/Y _0.5 h_	Up-regulated	XP_009344939.1	PREDICTED: phosphoinositide phospholipase C 2-like [*Pyrus x bretschneideri*]	0.236	3	2.186	0.005
Z _0.5 h_/Y_0.5 h_	Down-regulated	XP_009346269.1	PREDICTED: PI-PLC X domain-containing protein At5g67130-like [*Pyrus x bretschneideri*]	0.392	12	0.366	0.002
Z _0.5 h_/Y _control_	Up-regulated	XP_009373787.1	PREDICTED: PI-PLC X domain-containing protein At5g67130 [*Pyrus x bretschneideri*]	0.161	5	1.815	0.041
Z _0.5 h_/Y _control_	Up-regulated	XP_009344939.1	PREDICTED: phosphoinositide phospholipase C 2-like [*Pyrus x bretschneideri*]	0.236	3	1.603	0.036
Z _0.5 h_/Y _control_	Down-regulated	XP_009346269.1	PREDICTED: PI-PLC X domain-containing protein At5g67130-like [*Pyrus x bretschneideri*]	0.392	12	0.559	0.002
Y _0.5 h_/Z _control_	Up-regulated	XP_009346269.1	PREDICTED: PI-PLC X domain-containing protein At5g67130-like [*Pyrus x bretschneideri*]	0.392	12	1.308	0.002
Y _0.5 h_/Z _control_	Down-regulated	XP_009373787.1	PREDICTED: PI-PLC X domain-containing protein At5g67130 [*Pyrus x bretschneideri*]	0.161	5	0.814	0.003

*Q-value < = 0.05 and Foldchange > = 1.5 [Log2 (Ratio)] are set as the significant threshold for differentially expression. Y _*0.5 h*_ refers to that the styles of ‘Yali’ were collected at 0.5 h after self-pollination. Z _*0.5 h*_ refers to that the styles of ‘Jinzhuili’ were collected at 0.5 h after self-pollination. Z _*Control*_ refers to that the styles of ‘Jinzhuili’ were collected at 0.5 h without pollination.*

S-RNase inhibited *PLC* activity and arrested pollen tube growth. Furthermore, we cloned a gene fragment from *PI-PLC 2-like gene* (LOC103936790), and observed that the expression levels of the other two *PLC* genes (LOC103958605 and LOC103966250) were relatively higher after compatible pollination. According to proteomic analysis, there are other *PLC* genes involved in self-incompatibility. Therefore, it requires further research on the role of these *PLC* genes.

## Discussion

In Solanaceae, Plantaginaceae, and Rosaceae species, SI is genetically controlled by a single S-locus with multiple haplotypes. The pistil-S gene encodes a T2 family ribonuclease, namely S-RNase (Xue et al., 2014), but the identity of the pollen-S gene has not been confirmed. Current research suggests that the *SLF* (*S-locus F-box*) gene determines male specificity in SI. The *SLF* gene product forms SCF (SKP1/Cullin1/F-box) complexes that serve as putative E3 ubiquitin ligases, which interact with S-RNases and cause non-self-S-RNase degradation during compatible pollination (McClure, 2006). However, S-RNases from the style continuously enter pollen tubes. Immunolocalization studies showed an abundance of S-RNase inside compatible and incompatible *Solanum chacoense* pollen tubes (Luu et al., 2000). Furthermore, there was no evidence of S-RNase degradation by using ^3^H-labeled S_2_-RNase and *in vitro* grown *Nicotiana alata* pollen tubes (Gray et al., 1991). We were unable to determine the differences of S-RNase between pollen tubes treated with self-S-RNase and non-self-S-RNase by using a colloidal gold technique during *in vitro* experiments (Qu et al., 2016b). McClure (2006) proposed that S-RNase compartmentalization into vacuoles could restrict S-RNase cytotoxicity, which enabled compatible pollination. If the vacuoles ruptured and S-RNase was released into the cytoplasm, the resulting cytotoxicity would inhibit pollen tube growth during incompatible pollination. During the SI response in *Nicotiana* species, when the incompatible pollen tube growth was fully inhibited 6 days after pollination, the vacuolar membrane system was still intact in 80% of pollen tubes *in vivo* (Roldán et al., 2012). Therefore, the S-RNase degradation and S-RNase compartmentalization models cannot be used to fully explain the physiological function of the Pollen-S gene during GSI responses. It is noteworthy that even though Rosaceae species also employ S-RNase and SLF as the female and male determinants respectively, the role of SLF in SI has not been established. The Japanese pear has a ‘non-self-recognition by multiple factors’ SI system, while a ‘self-recognition by a single factor’ system exists in *Prunus* species (Kakui et al., 2011).

The quantities of S-proteins differ among cultivars. However, they do not correlate with the growth of selfed pollen tubes. For example, there are much more S_4_-proteins in ‘Nijisseiki’ and ‘Kikusui’ than in ‘Yakumo,’ even though ‘Nijisseiki’ and ‘Kikusui’ exhibit intermediate levels of SI, and ‘Yakumo’ experiences strong SI (Zhang and Hiratsuka, 1999). Qin et al. (2006) observed that in accordance with the average values of S_11_- and S_12_-RNase for a single *Solanum chacoense* style, the levels of the S_11_- and S_12_-RNases can differ by up to 10-fold within a genotype. Additionally, the S_12_-RNase levels can differ by over threefold when different genotypes are compared. Surprisingly, the abundance of S_12_-RNase in different styles of the same plant can differ by over 20-fold. Therefore, S-RNase is not only responsible for just degrading RNA during the self-incompatible response. Early grafting experiments conducted by Lush and Clarke (1997) showed that growth inhibition caused by incompatible styles could be reversed in some pollen tubes, suggesting that the cytotoxic effect of S-RNase was not only caused by its role in RNA degradation.

The S-RNase protein structure is consistent with a dual role as both a cytotoxin and a recognition protein (McClure, 2006). Recently, several new functions of S-like RNases unrelated to ribonuclease activity have been uncovered, indicating that their cytotoxicity may be a result of their effects on proteins rather than RNA (Levava et al., 2006; Zhang et al., 2009; Luhtala and Parker, 2010). S-RNases modify the actins in the cytoskeleton of self-pollen tubes *in vitro*. These modifications occur prior to the arrest of pollen tube growth. The S-RNases may initiate programmed cell death during self-pollen tube growth inhibition *in vitro* in *P. pyrifolia* GSI responses (Liu et al., 2007; Wang et al., 2010).

Phosphoinositides are most likely present in every plant cell, but their most prominent function is pollen development and pollen tube polarity (Heilmann and Ischebeck, 2016). Phosphatidylinositol-specific phospholipase C controls pollen tube tip growth by regulating the levels of its substrate PIP_2_ and its cleavage product IP_3_ (Franklin-Tong et al., 1996; Zonia and Munnik, 2004; Monteiro et al., 2005). The maintenance of a Ca^2+^ gradient and vesicle secretion in pollen tube apices is essential for growth. Phosphatidic acid, PIP_2_, and IP_3_ play vital roles in the regulation of these processes (Monteiro et al., 2005; Krinke et al., 2007). Helling et al. (2006) did not study the relationship between Ca^2+^ and *PI-PLC* in pollen tube apices, but they proposed that IP_3_ generated at the flanks of the pollen tube tips during hydrolysis of PIP_2_ by *PI-PLC* may help establish the tip-focused cytoplasmic Ca^2+^ gradient essential for the growth of these cells. Moreover, Kost et al. (1999) also hypothesized that *PLC*-mediated hydrolysis of tip-localized PIP_2_ and IP_3_-induced Ca^2+^ influx into the cytoplasm may be involved in the establishment of the tip-focused Ca^2+^ gradient. Additionally, the putative IP_3_-regulated Ca^2+^ channels, which allow Ca^2+^ to enter from the extracellular matrix, may be present in the pollen tube plasma membrane. However, IP_3_-sensitive Ca^2+^ channels have not been identified in plants (Munnik et al., 1998; Meijer and Munnik, 2003). Dellis et al. (2006) believed that the IP_3_ receptors (IP_3_R) contribute more directly to Ca^2+^ entry across the plasma membrane. In the present study, we demonstrated that hyperpolarization-activated Ca^2+^ channels in the plasma membrane of *P. pyrifolia* pollen tube apices were sensitive to IP_3_. When IP_3_ was added to pipette solutions, the Ca^2+^ currents presented significant increases, or they were recovered if they had been suppressed by U-73122. Thus, we suggest that active *PLC* located at the pollen tube tips cleaves PIP_2_ to IP_3_, which then increases the extracellular Ca^2+^ influx through hyperpolarization-activated Ca^2+^ channels. Ca^2+^ influx at the apices of pollen tubes not only maintains the Ca^2+^ gradient from tip to base, but also stimulates pollen tube elongation.

Although it is difficult to assess direct correlations among ion flux, ion gradients, delivery and turnover of vesicles, and pollen growth (Messerli et al., 1999), there is no doubt they are closely linked (Blakeslee et al., 2004). The growing pollen tube tip is filled with secretory vesicles that fuse with the membrane to drive pollen tube elongation (Hepler, 2015). Campanoni and Blatt (2007) suggested that exocytosis from the Golgi apparatus enriched the ion channels at the tip of growing pollen tubes through site-directed exocytosis or lateral diffusion. We speculated that Ca^2+^ channel on the membrane maintains the sensitivity of IP_3_. Moreover, based on transmission electron microscopy, we observed that a large number of vesicles were fusing with the cell membrane at the tip of pollen tube (data unpublished). According to patch clamp technique, IP_3_ regulates the extracellular calcium influx through the Ca^2+^ channel within the cytoplasmic membrane (Vaca and Kunze, 1995; Dellis et al., 2006). These results are consistent with our findings that IP_3_ can increase extracellular calcium influx at apical pollen tube.

The *PI-PLC* activity assay showed that self-S-RNase (‘Housui’) decreased the activity of *PLC* extracted from ‘Housui’ pollen tubes. In the pollen tube, the *PLC* gene was inserted into the mutant, which resulted in the conversion from the self-incompatible ‘Yali’ cultivar to the self-compatible ‘Jinzhuili’ cultivar. The *PLC* only interacted with one S-RNase according to yeast two hybrid technology in ‘Dangshansuli,’ but if the *PLC* insertion mutation is artificially performed, the mutated *PLC* could not interact with any S-RNase. These results were consistent with the inhibition model proposed by Wu et al. (2012a) in their research. The amino acid sequences of pollen *PLC* have polymorphisms that may match the S-RNase polymorphisms in the style. Moreover, IP_3_ can enable full recovery of Ca^2+^ currents following suppression by self-S-RNase or U-73122. Thus, self-S-RNase selectively blocks the activity of a pollen-specific *PLC* located in the apical plasma membrane of pollen tubes, and decreases Ca^2+^ influx through hyperpolarization-activated Ca^2+^ channels. This ultimately leads to a decrease or elimination of the Ca^2+^ gradient.

The transcriptomic analyses of pollen–pistil interactions have provided a valuable approach and a wealth of information on which to base future functional analyses (Dresselhaus and Franklin-Tong, 2013). According to the pathway enrichment analysis of differentially expressed proteins based on KEGG database, the pathway of plant–pathogen interaction was significant after pollination (Supplementary Figure S4). Therefore, we suggested that the *PLC* activity in pollen tube was selectively blocked by self-S-RNase during GSI in *P. pyrifolia*, but the process was very slow due to abundance of S-RNase (about 20 ng) in *P. pyrifolia* styles (Zhang and Hiratsuka, 2005). The Ca^2+^ gradient was gradually eliminated, and the growth rate of the incompatible pollen tube decreased, but it was not completely arrested (Herrero and Dickinson, 1980; Lush and Clarke, 1997). Roldán et al. (2012) also suggested that the inhibition of pollen tube growth was a progressive process rather than a rapid and uncontrolled collapse of cellular structures. As the incompatible pollen tube growth slowed down, S-RNase accumulated and began to degrade RNA when it exceeded the threshold concentration in the pollen tube, which caused further arrest. This is consistent with the dependence of S-haplotype-specific pollen rejection on S-RNase concentration (Hiratsuka et al., 2001; Takayama and Isogai, 2005).

Many studies on the GSI of Rosaceae focused the fate of pollen tubes within the style, while ignoring the viability of pollen in the stigma (Romain and Dirk, 2014). Furthermore, no studies have so far examined the changes in gene expression that accompany mutual interactions between pollen and stigma (Kao and Huang, 1994). In this study, we investigated the changes in gene expression that occur in the gametophyte during early stage after self-compatible and self-incompatible pollinations in *Pyrus*. At the early stage of pollination, under incompatible pollination, the expression of PLC gene in pollen tube was lower than that under compatible pollination. The low expression of *PLC* gene may be related to the slow growth of pollen tubes in the style, but what factors induce the low expression of *PLC* gene need further exploration. Vossen et al. (2010) believed that *PLC* isoforms in plant were required for the hypersensitive response (HR) and disease resistance. However, there is a differential requirement of *PLC* isoforms for the tomato [*Solanum lycopersicum* (*SI*)] immune response, and *SIPLC*4 is specifically required for Cf-4 function, while *SIPLC*6 may be a more general component of resistance protein signaling. We found that the expression of *PLC* isoforms was significantly increased under compatible pollination according to the results of iTRAQ. Perhaps these *PLCs* are involved in self-incompatibility, but the specific role may not be the same.

**FIGURE 10:**
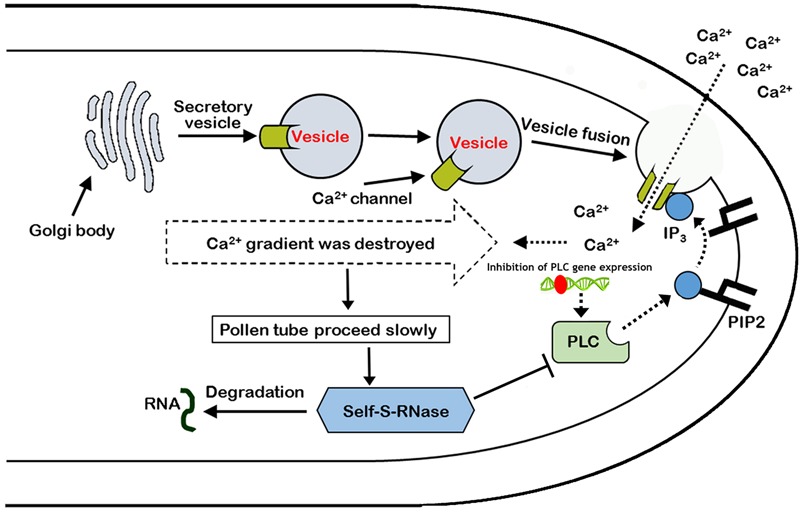
Model of the arrest of pollen-tube growth by self-S-RNase in *Pyrus pyrifolia*. *PLC* in pollen tube, located at pollen tube apices, cleaves PIP_2_ to IP_3_ and DAG (1,2-Diacylglycerol). Inositol 1,4,5-trisphosphate stimulates extracellular free-Ca^2+^ influx through Ca^2+^ channels, maintaining the Ca^2+^ gradient. If the activity of the special apical tube *PLC* is blocked by self-S-RNase, the concentration of IP_3_ decreases at the apex. In addition to the inhibition of *PLC* activity, the expression of *PLC* gene was also inhibited by unknown factors. As a result, the activity of Ca^2+^ channels is suppressed, and extracellular free-Ca^2+^ influx decreases. Additionally, the high Ca^2+^ gradient at the tip cannot be generated and maintained (Campanoni and Blatt, 2007). Therefore, the pollen tube eventually stops growing. Roldán et al. (2012) concluded that the style rejected incompatible pollen through S-RNase degrading the RNA, which was shown to be essential for the SI response, but the procession is at the late stage after major cellular dismantling of incompatible pollen tubes. In this self-incompatible model, the *PLC* activity and expression in the non-compatible pollen tube coincide with the behavior of the *PLC* between plant and pathogen. Type T symbol represents inhibition of PLC enzyme activity. Broken and solid lines represent negative and positive regulatory activities, respectively. Red ellipse represents an unknown factor that inhibits *PLC* gene expression.

In summary, it was found that self-S-RNase inhibited the activity of *PLC* enzyme, and there was a positive correlation between the growth of pollen tube and the expression of PLC gene during the process of mutual recognition between pollen and stigma. Moreover, self-S-RNase decreased the activity of Ca^2+^ channels and disrupted the Ca^2+^ gradient at the tip of the growing pollen tube during the GSI response through *PLC-IP_3_* single pathway. ‘Jinzhuili’ is a naturally occurring SC mutant from SI of ‘Yali,’ because the S-RNase cannot recognize the insertion of mutated *PLC* in the pollen tube. Thus, we suggested that *PLC* in the pollen tube is one of the determinant male specificity factors during SI interactions. We proposed a new model to explain these results (**Figure 10**).

## Materials and Methods

### Samples

We used two pear varieties of ‘Housui’ and ‘Imamuraaki’ to investigate GSI in *P. pyrifolia*. The S-genotypes of ‘Housui’ and ‘Imamuraaki’ are *S_3_S_5_* and *S_1_S_6_*, respectively (Ishimizu et al., 1998). The two varieties are completely self-incompatible after self-pollination, but show strong compatibility when cross-pollinated.

### Pollen Collection, S-RNase Extraction, and Isolation of Protoplasts from Pollen Tubes

We collected ‘Housui’ (*P. pyrifolia* Nakai) pollen from blooming flowers in a fruit experimental garden in Jiangsu, China. The collected pollen was stored at -20°C. S-RNase was extracted from ‘Housui’ and ‘Imamuraaki’ styles according to previously described methods (Hiratsuka et al., 2001). The final S-RNase concentration was 1.0 μg μl^-1^ with 0.15 units of activity in the medium. The medium composed of 0.55 mM Ca(NO_3_)_2_, 1.6 mM H_3_BO_3_, 1.6 mM MgSO_4_, 1 mM KNO_3_, 440 mM sucrose and 5 mM 5, 2-(*N*-Morpholino) ethane sulfonic acid hydrate (MES), and pH was adjusted to pH 6.0 with Tris. Protoplasts were isolated from pollen tube apices according to our established method (Qu et al., 2007).

### Pollen Tube Staining with fluo-4/AM

Pollen tubes were cultured for 2.0 h, and then stained with 1 μM fluo-4/AM dye (Dôjindo Laboratories) for 15 min at room temperature in the dark according to our methods (Qu et al., 2016a). We used a TCS SP-2 MP AOBS Confocal System (Leica) to observe the tubes. The fluorescence intensity was measured with Image Pro Plus software (Qu et al., 2016a).

### Electrophysiology and Data Analysis

Pipettes were pulled from borosilicate glass blanks and coated with Sylgard (184 Silicone Elastomer kit; Dow Corning). Pipettes with solution had resistance values ranging from 15 to 35 MΩ in 10 mM CaCl_2_. Whole-cell currents across the plasma membrane of protoplasts isolated from pollen tubes were measured with an Axon 200B amplifier (Axon Instruments). Whole-cell preparations were obtained by forming a giga seal in the cell-attached mode, and the membrane was ruptured with a short burst of extra suction. A substantial increase in capacitance indicated that the whole-cell configuration was achieved, and the series resistance and capacitance were adjusted accordingly. Voltage-pulses of 2.5 s were used to elicit voltage-dependent currents. Data were sampled at 2 kHz and filtered at 0.5 kHz. Records were stored and analyzed by using pClamp 9.0 (Axon Instruments). The junction potential was corrected according to a published report (Amtmann and Sanders, 1997). All experiments were conducted at room temperature (20–22°C). The current-voltage curves were constructed by using the current values measured at the final voltage.

### Experimental Solutions

The basal solution consisted of 10 mM CaCl_2_, 0.2 mM glucose, and 0.05 mM MES. The bath solutions were adjusted to 800 mOsM and pH 5.8 with mannitol and Tris. The internal basal pipette solution contained 1 mM MgCl_2_, 0.1 mM CaCl_2_, 4 mM Ca(OH)_2_, 10 mM EGTA, 2 mM MgATP, 10 mM HEPES, 100 mM CsCl, and 0.1 mM GTP, with pH 7.3. The solution was adjusted to 1,100 mOsM with mannitol and Tris. The free Ca^2+^ concentration of the pipette solution was approximately 10 nM. Details regarding the bath and pipette solutions are provided in the figure legends.

### Preparation of Plasma Membranes

Mature pollen grains (0.1 g, approximately 1 × 10^7^ cells ml^-1^) were suspended in 10 ml medium and cultured for 3 h at 25°C. Subsequently, pollen tubes were washed with 0.8 M sorbitol and quickly frozen with liquid nitrogen. The tubes were then homogenized in 20 mM Hepes-Tris (pH 7.8), 1 mM EGTA (pH 8.0), 0.25 mM sorbitol, 0.5% (w/v) BSA, 1 mM PMSF, 5 mM DTT, and 0.7% PVPP for membrane isolation. Tissue debris was removed by centrifugation at 10,000 × *g* for 20 min at 4°C. The supernatant was further centrifuged at 100,000 × *g* for 60 min at 4°C for microsomal membrane preparation. Highly purified plasma membranes were prepared from the pellet through two-phase partitioning (Tate and Gruner, 1989). All steps were performed at 4°C. Protein concentrations were measured by using a bicinchoninic acid protein assay reagent with BSA as a standard (Smith et al., 1985). The cell membrane extracted from the experimental procedure can be used to evaluate the activity of phospholipase C (Liu et al., 2006).

### *PI–PLC* Activity Assay

Phospholipase C activity was assayed according to a published method (Helling et al., 2006). The standard reaction mixture contained 50 mM Tris-maleate (pH 6.0), 0.8 mM Ca^2+^/EGTA, 0.08% (w/v) sodium deoxycholate, 10 μl self-S-RNase (‘Housui’) or non-self-S-RNase (‘Imamuraaki’), 0.74 kBq [^3^H]PIP_2_ (251.6 GBq mmol^-1^, head group labeled; Perkin-Elmer), and 10 μg membrane protein in a final volume of 50 μl. The reaction was initiated by adding micellar substrate solutions, and stopped after 20 min at 25°C by adding 1 ml chloroform: methanol (2: 1, v/v) and 250 μl 1 M HC1. After vortexing and a brief centrifugation, the radioactive reaction products were recovered in the upper phase, and quantified with a liquid scintillation counter (LS 6500 Multi-Purpose Scintillation Counter; Beckman Coulter). Background radioactivity detected in control samples incubated without protein was subtracted from all data.

### Yeast Two-Hybrid Interaction Assays

The diploid *P. x bretschneideri* Rehd. is the first pear species with its genome comprehensively sequenced (Wu et al., 2012b). We compared the sequence of our cloned *PLC* cDNA fragments isolated from pollen tubes with that of the ‘DangshanSuli’ (*P. x bretshneideri* Rehd.) genome to determine the full-length *PLC* sequence. The full-length cDNA sequences of *S_7_-RNase* and *S_34_-RNase* (DQ414813) were based on the *P. x bretschneideri* Rehd. genome and GenBank sequences, respectively. The *S_7_-RNase* and *S_34_-RNase* cDNA sequences were separately cloned into the pGADT7 vector (Clontech). The *PLC* cDNA sequence was cloned into the pGBKT7 vector (Clontech). Comparing with the *PLC* gene mutation of the ‘Jinzhuili,’ we inserted 78 nucleotide residues, which encoded 26 amino acid residues, into the same mutant position in the ‘Dangshansuli’ *PLC* gene. The mutation of the ‘Dangshansuli’ *PLC* gene was also cloned into the pGBKT7 vector (Clontech). Each bait/prey pair was introduced to the AH109 yeast strain (Clontech). As a control for auto-activation false-positives, each bait was also co-transformed into the yeast strain with an empty AD vector, and each prey was co-transformed with an empty BD vector. The bait/prey pair colonies that grew on all selective media (Trp-Leu and Trp-Leu-Ade-His) were considered positive for interaction.

### Microscopy and Live-Cell Imaging

For Tobacco leaves expressing PLC-GFP and S-RNase-RFP, fluorescence localization and image processing were performed as described in Francinallami et al. (2011) and Zhong et al. (2012).

### Preparation of RNA Samples

Total RNA was extracted from ‘Housui’ and ‘Imamuraaki’ pollen grains as previously described (Tao et al., 1999), and treated with DNase I (Invitrogen). The integrity of the RNA was checked by electrophoresis, and concentrations were determined by spectrophotometry. Total RNA (1 μg) was used for first-strand cDNA synthesis with the Micro-FastTrack 2.0 mRNA Isolation Kit (Invitrogen) by following the manufacturer’s instructions. The cDNA then served as templates for subsequent polymerase chain reaction (PCR) amplifications.

### Reverse Transcription-PCR Analysis of *PI-PLC* in ‘Housui’ and ‘Imamuraaki’ Cultivars

We reverse-transcribed mRNA to synthesize first-stand cDNA by using the adapter primer NotI-(dT)18. The 3′-region of cDNA encoding *PI-PLC* was amplified with NotI-(dT)18 and a forward primer (5′-CAGCTKAGYAGYGAYTGYAGTG-3′) designed based on the X-domain region. The 5′-ends of the cDNA were amplified by using the 5′-RACE System 2.0 (Invitrogen) with reverse primers (5′-TGAACATWCCDTGCATRAACCAAAG-3′ and 5′-TNAGDATYTTCCGCTGAGTYAACCTG-3′) specific for *PI-PLC*. The partial-length cDNA encoding *PI-PLC* was obtained through 3′ and 5′ RACE cloning.

### High Throughput Analysis of Differential Gene Expression

‘Jinzhuili’ is a spontaneous self-compatible mutant of ‘Yali’ (*P. x bretschneideri* Rehd., *S_21_S_34_*). ‘Yali’ is a major Chinese pear cultivar, which displays a typical S-RNase-based GSI (Wu et al., 2013). We collected the styles from 100 flowers of each cultivar 0.5 h after self-pollination. We used non-pollinated ‘Jinzhuili’ styles as controls. Total RNA was extracted from each sample with Takara reagent (Takara Bio) by following the manufacturer’s protocol. RNA quantity, purity, and quality were analyzed with a spectrophotometer (SMA4000 UV-VIS spectrophotometer; Varian, Inc.). Double-stranded cDNA libraries were then constructed by using the TruSeq RNA Sample Preparation kit v2 (Illumina). Each sample pool was sequenced on the MiSeq (Illumina) platform with sequence runs of 2 × 250 paired-end reads at The Central Facility of the Institute for Molecular Biology and Biotechnology, McMaster University, Canada.

The *P. pyrifolia* reference genome and annotation data were obtained from GenBank^2^. The transcriptome genome coverage was deduced based on our transcriptome data (4.0 Gb) and the *P. x bretschneideri* Rehd. genome data (432 Mb) from the Centre of Pear Engineering Technology Research, State Key Laboratory of Crop Genetics and Germplasm Enhancement (Wu et al., 2012b).

In our project, we sequenced three samples on the Illumina HiSeq platform. On average, we generated about 4.46 Gb bases from each sample. After mapping sequenced reads to the reference genome and reconstructing transcripts, we obtained 7,251 novel transcripts from all samples. Among them, 4,997 were previously unknown splicing variants from known genes, 999 were novel coding transcripts without any known features, and the remaining 1,255 were long non-coding RNA. The sequencing reads containing low quality, adaptor polluted and high content of unknown bases (N) were removed before downstream analyses. After filtering, the read-quality metrics were generated (Supplementary Table S3). The filtered reads were mapped to the reference genome by using HISAT (Kim et al., 2015). On average, 59.79% of the reads were mapped, and the uniformity of the mapping results between samples suggested that the results of these analyses were comparable. The mapping details are summarized in Supplementary Table S4.

### GO Analysis

Gene ontology (GO) analysis was performed to determine the biological implications of the expression of unique genes in significant or representative profiles of genes that were differentially expressed. We downloaded GO annotations from NCBI^3^, UniProt^4^, and the Gene Ontology Consortium^5^. Fisher’s exact test was conducted to identify significant GO categories, and false discovery rate (FDR) was used to correct the *p*-values (Dupuy et al., 2007).

### Pathway Analysis

Pathway analysis was used to determine the significant pathway(s) of the DEGs based on the KEGG database. Fisher’s exact test was conducted to select significant pathways, and the threshold of significance was defined according to the *p*-value and FDR (Kanehisa et al., 2004; Yi et al., 2006; Draghici et al., 2007).

### Quantitative Real-Time RT-PCR to Determine Gene Expression in Style

To estimate mRNA expression levels of *PLC* genes in style after compatible and incompatible pollination, qRT-PCR analysis was performed. After 0.5 h of artificial pollination, we collected the styles and immediately placed them into liquid nitrogen for preservation. Total RNA was extracted with TRIzol reagent (Invitrogen), and the first-strand cDNA was synthesized from 2 μg of total RNA with an Omniscript RT kit (Qiagen). The primers used for RT-PCR are shown in Supplementary Table S2. Actin gene was used as the reference gene. All quantitative RT–PCR experiments were performed in biological triplicate and technical duplicate. Relative quantification was normalized to the housekeeping control gene ubiquitin, and FC (fold-change) in gene expression was calculated with the 2^-ΔΔ^*^C^*^T^ method (Livak and Schmittgen, 2001).

### Proteome Analysis

The three samples used in high-throughput sequencing were used to perform the proteome analysis. The total protein of each corresponding group was blocked, digested and labeled according to the iTRAQ protocol (BGI, Co., Ltd, China). The labeling was as follows: ‘Yali’ 0.5 h (113), ‘Jinzhuili’ Control (114) and ‘Jinzhuili’ 0.5 h (115). All LC-ESI-MSMS analysis was based on the Triple TOF 5600 system (SCIEX, Framingham, MA, United States) fitted with a Nanospray III source (SCIEX, Framingham, MA, United States), and a pulled quartz tip as the emitter (New Objectives, Woburn, MA, United States). The *IQuant* software was used to conduct protein identification, tag impurity correction, data normalization, missing value imputation, protein ratio calculation, statistical analysis, and results presentation for iTRAQ (Wen et al., 2014). The three samples went through a batch of machine tests. No difference in the number of proteins was identified between samples, and no abnormal sample was found. The labeling efficiency and label quality after mass spectrometry were subject to quality-control tests. The ratios of the medians of tag intensities were close to 1. The iTRAQ labeling efficiency was 96.44%. Protein identification was based on unique peptide segments with its threshold set at a minimum of one unique peptide segment. The peptide segments retained for quantification were the segments that uniquely identified individual proteins. The search was performed against *P. x bretschneideri* Rhd. genome data.

## Author Contributions

HQ wrote paper and designed and analyzed the experiments. YG performed the experiments. YW designed the experiment and collected the pollen and done the physiology experiments and revised the manuscript. SZ designed the part of experiments. All of the authors analyzed the results and approved the final version of the manuscript.

## Conflict of Interest Statement

The authors declare that the research was conducted in the absence of any commercial or financial relationships that could be construed as a potential conflict of interest.

## Footnotes

1 http://www.ncbi.nlm.nih.gov/gorf/gorf.html

2 http://www.ncbi.nlm.nih.gov/nuccore/

3 http://www.ncbi.nlm.nih.gov/

4 http://www.uniprot.org/

5 http://www.geneontology.org/
