# Supplementary material for: PLC-Mediated Signaling Pathway in Pollen Tubes Regulates the Gametophytic Self-incompatibility of Pyrus Species
Source: Front Plant Sci. 2017 Jul 6;8:1164. doi: 10.3389/fpls.2017.01164 (PMC5498517; doi:10.3389/fpls.2017.01164)
Supplement: Supplementary file 1 [file Table_1.pdf]

Supplementary Table S1. The differences in the expression of 15 PLC genes and their definitions.

Serial number	GeneID	$Log_2(Z_{0.5h}/Z_{CK})$	$Log_2(Z_{0.5h}/Y_{0.5h})$	$Log_2(Y_{0.5h}/Z_{CK})$	Definition
1	LOC103938930	0.55	-1.26	0.80	phosphoinositide phospholipase C 4-like
2	LOC103938929	-0.49	-0.67	0.68	phosphoinositide phospholipase C 2-like
3	LOC103958607	-0.11	1.01	-1.13	phosphoinositide phospholipase C 2-like
4	LOC103958605	2.69	2.86	0.10	phosphoinositide phospholipase C 6-like
5	LOC103936790	4.56	6.28	none	phosphoinositide phospholipase C 2-like
6	LOC103936781	0.72	-1.20	0.16	phosphoinositide phospholipase C 6-like
7	LOC103956577	none	-1.70	0.19	phosphatidylglycerol phospholipase C
8	LOC103938348	-0.31	1.10	-1.42	PI-PLC X-box domain-containing protein DDB_G0293730-like
9	LOC103932394	none	none	none	PI-PLC X-box domain-containing protein DDB_G0293730-like
10	LOC103932386	none	none	none	PI-PLC X-box domain-containing protein DDB_G0293730-like
11	LOC103930336	none	none	none	PI-PLC X-box domain-containing protein DDB_G0293730-like
12	LOC103930332	none	none	none	PI-PLC X-box domain-containing protein DDB_G0293730-like
13	LOC103966250	6.67	6.40	none	PI-PLC X-box domain-containing protein DDB_G0293730-like
14	LOC103966241	none	none	none	PI-PLC X-box domain-containing protein DDB_G0293730-like
15	LOC103958481	9.32	-1.12	10.44	PI-PLC X-box domain-containing protein DDB_G0293730-like

Note:

(1) $Y_{0.5h}$ is that the styles of ‘Yali’ were collected at 0.5 h after self-pollination. $Z_{0.5h}$ is that the styles of ‘Jinzhuili’ were collected at 0.5 h after self-pollination. Z_{CK} is that the styles of ‘Jinzhuili’ were collected at 0.5 h without pollination.

(2) Positive values represent up-regulated gene and Negative values represent the down regulated genes. None: This gene was not found in the list of differences according to high-throughput sequencing.

(3) We need to highlight the genes with red font in the table, and carry out quantitative PCR.
