# Supplementary material for: PLC-Mediated Signaling Pathway in Pollen Tubes Regulates the Gametophytic Self-incompatibility of Pyrus Species
Source: Front Plant Sci. 2017 Jul 6;8:1164. doi: 10.3389/fpls.2017.01164 (PMC5498517; doi:10.3389/fpls.2017.01164)
Supplement: Supplementary file 2 [file Table_2.PDF]

Supplementary Table S2. Primers used for Quantitative PCR analysis. Actin gene was used as reference gene.

GeneID	Primer set	
	Forward primer (5’-3’)	Reverse primer (5’-3’)
LOC103958605	ACTCAGTAGTGACTGCAGTG	AGCATTTGATTAGCGTCACAG
LOC103936790	GTGCACCATGATATGAATGCG	ATGAAGCACATCGACATCGTC
LOC103966250	ACACTGTGTGATCTTCACCAG	ATGCATGGAACTCCAATCTTG
LOC103932386	ATCAGCTCGGCGAGTTTTTA	GAATTTCGTGGATGGCAAGT
Actin	TGGTGTCATGGTTGGTATGG	CAGGAGCAACACGAAGTTCA
