# Supplementary material for: PLC-Mediated Signaling Pathway in Pollen Tubes Regulates the Gametophytic Self-incompatibility of Pyrus Species
Source: Front Plant Sci. 2017 Jul 6;8:1164. doi: 10.3389/fpls.2017.01164 (PMC5498517; doi:10.3389/fpls.2017.01164)
Supplement: Supplementary file 3 [file Table_3.pdf]

Supplementary Table S3. Assembled and annotated transcriptomes from self-compatible and self-incompatible styles (JZ_0.5h and YL_0.5h, respectively) and styles without pollination as control (JZ_CK).

Sample	Total Raw Reads (Mb)	Total Clean Reads (Mb)	Total Clean Bases (Gb)	Clean Reads Q20(%)	Clean Reads Q30(%)	Clean Reads Ratio(%)
JZ_CK	35.05	29.73	4.46	98.87	96.42	84.82
JZ_0.5h	35.05	29.91	4.49	98.85	96.36	85.31
YL_0.5h	35.05	29.55	4.43	98.96	96.61	84.29

Q20: the rate of bases which quality is greater than 20.
