# Supplementary material for: PLC-Mediated Signaling Pathway in Pollen Tubes Regulates the Gametophytic Self-incompatibility of Pyrus Species
Source: Front Plant Sci. 2017 Jul 6;8:1164. doi: 10.3389/fpls.2017.01164 (PMC5498517; doi:10.3389/fpls.2017.01164)
Supplement: Supplementary file 4 [file Table_4.pdf]

Supplementary Table S4. Summary of Genome Mapping

Sample	Total CleanReads	Total MappingRatio	Uniquely MappingRatio
jinzhui1A	29,732,362	60.80%	52.37%
jinzhui2A	29,905,934	61.02%	52.38%
yali2A	29,547,452	57.55%	49.25%

Uniquely Mapping: Reads that map to only one location of reference, called uniquely mapping.
