# Supplementary material for: PLC-Mediated Signaling Pathway in Pollen Tubes Regulates the Gametophytic Self-incompatibility of Pyrus Species
Source: Front Plant Sci. 2017 Jul 6;8:1164. doi: 10.3389/fpls.2017.01164 (PMC5498517; doi:10.3389/fpls.2017.01164)
Supplement: Supplementary file 5 [file Image_1.pdf]

Supplementary figure S1. The expression of PLC, S7-RNase, S34-RNase and PLC-MU (insert the mutated PLC) proteins in yeast cells was detected by Western blot. The experimental group PGBKT7/PLC could detect the expression of BD-PLC fusion protein at 65KD level. The expression of AD-S7-RNase fusion protein could be detected at the level of 44KD in the experimental group transformed with PGADT7 / S7-RNase. The expression of AD-S34-RNase fusion protein could be detected at the 44KD level in the experimental group of S34-RNase. The experimental group PGBKT7/PLC-MU could detect the expression of BD-PLC fusion protein at 65KD level. Lane 1: PGBKT7/PLC; Lane 2: PGADT7/S7-RNase; Lane 3: PGADT7/S34-RNase; PLC-MU: PGBKT7/PLC-MU.

Amounts of protein loaded: Total protein concentration of yeast lysis was around 60 μg/μL, 20 μL sample of yeast lysis was loaded on SDS-GEL. **Antibodies used for detection:** Rabbit anti-c-Myc Monoclonal Antibody (Article numbe: ZA-0555. Zsbio commerce store, Beijing, China). **Loading control:** The WB test was just aimed to confirm the expression of target protein in yeast, so loading cortrol is not necessary in this experiment. But we did load the sample of pGBKT7 vector only as control to make sure the antibody works well, the result was in the attachment. (molecular weight of BD domain: 20KD)

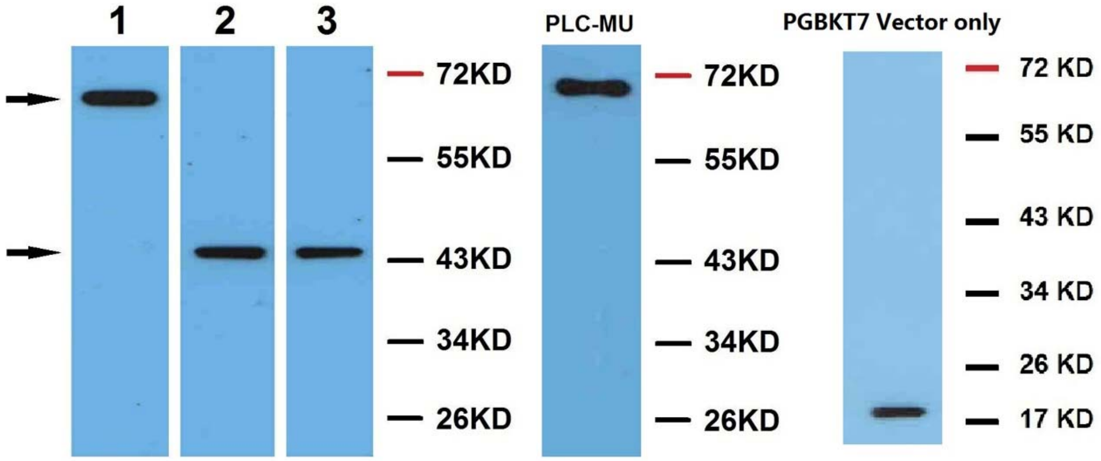
1
2
3
72KD
55KD
43KD
34KD
26KD
PLC-MU
72KD
55KD
43KD
34KD
26KD
PGBKT7 Vector only
72 KD
55 KD
43 KD
34 KD
26 KD
17 KD
