# Supplementary material for: PLC-Mediated Signaling Pathway in Pollen Tubes Regulates the Gametophytic Self-incompatibility of Pyrus Species
Source: Front Plant Sci. 2017 Jul 6;8:1164. doi: 10.3389/fpls.2017.01164 (PMC5498517; doi:10.3389/fpls.2017.01164)
Supplement: Supplementary file 6 [file Image_2.pdf]

Supplementary figure S2. 'Jinzhuili' and 'Yali' cross pollination and self-pollination.

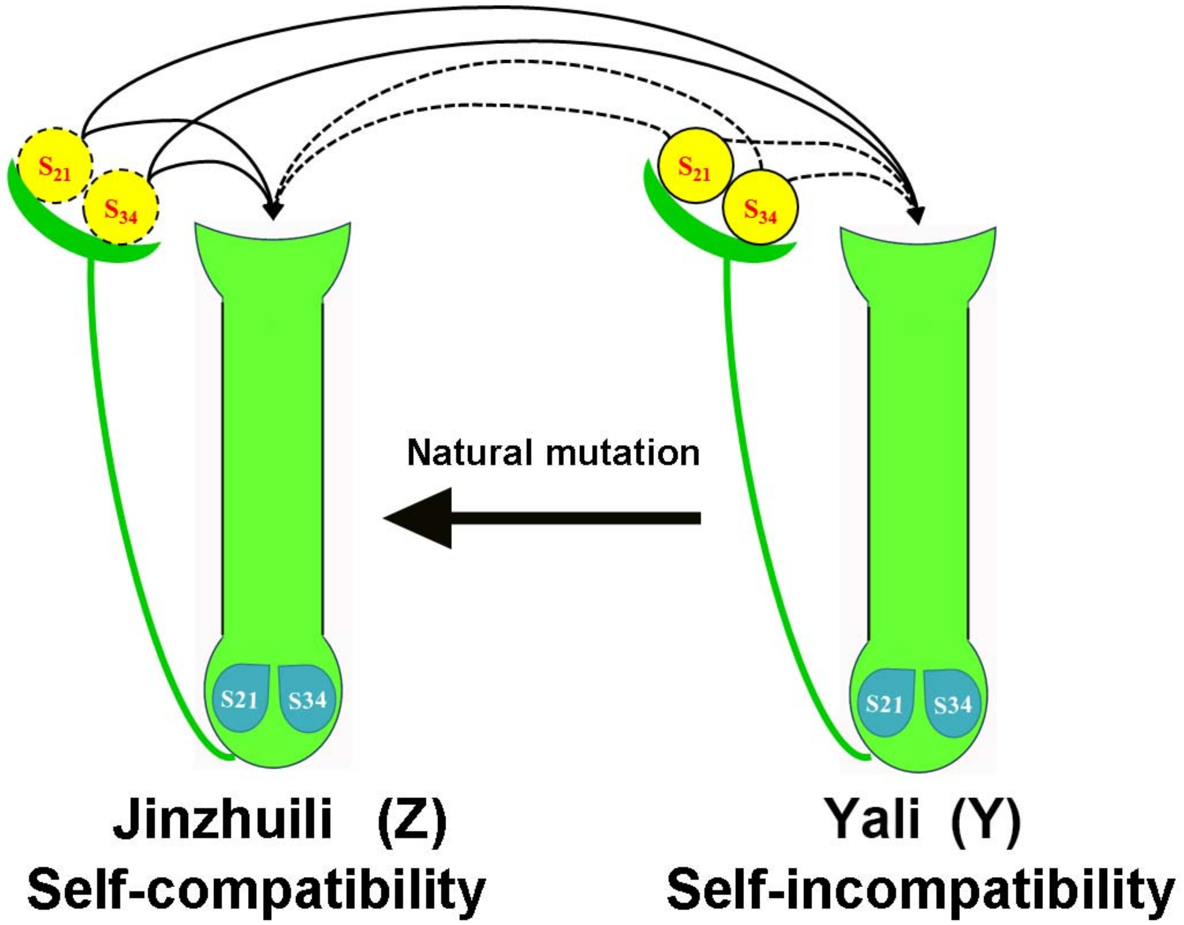

Note: Solid line indicates compatible pollination and dotted line indicates the incompatible pollination.
