# Supplementary material for: PLC-Mediated Signaling Pathway in Pollen Tubes Regulates the Gametophytic Self-incompatibility of Pyrus Species
Source: Front Plant Sci. 2017 Jul 6;8:1164. doi: 10.3389/fpls.2017.01164 (PMC5498517; doi:10.3389/fpls.2017.01164)

Supplementary figure S3. The pollen grows in the style at 0.5 h after pollination. At 0.5 h after artificial pollination, no matter whether they were pollinated by a compatible or an incompatible pollination, the pollen just germinated on the stigma, and there was no difference in pollen tube growth. (a) cross-pollination; (b) self-pollination. Scar bar is 200 μm.

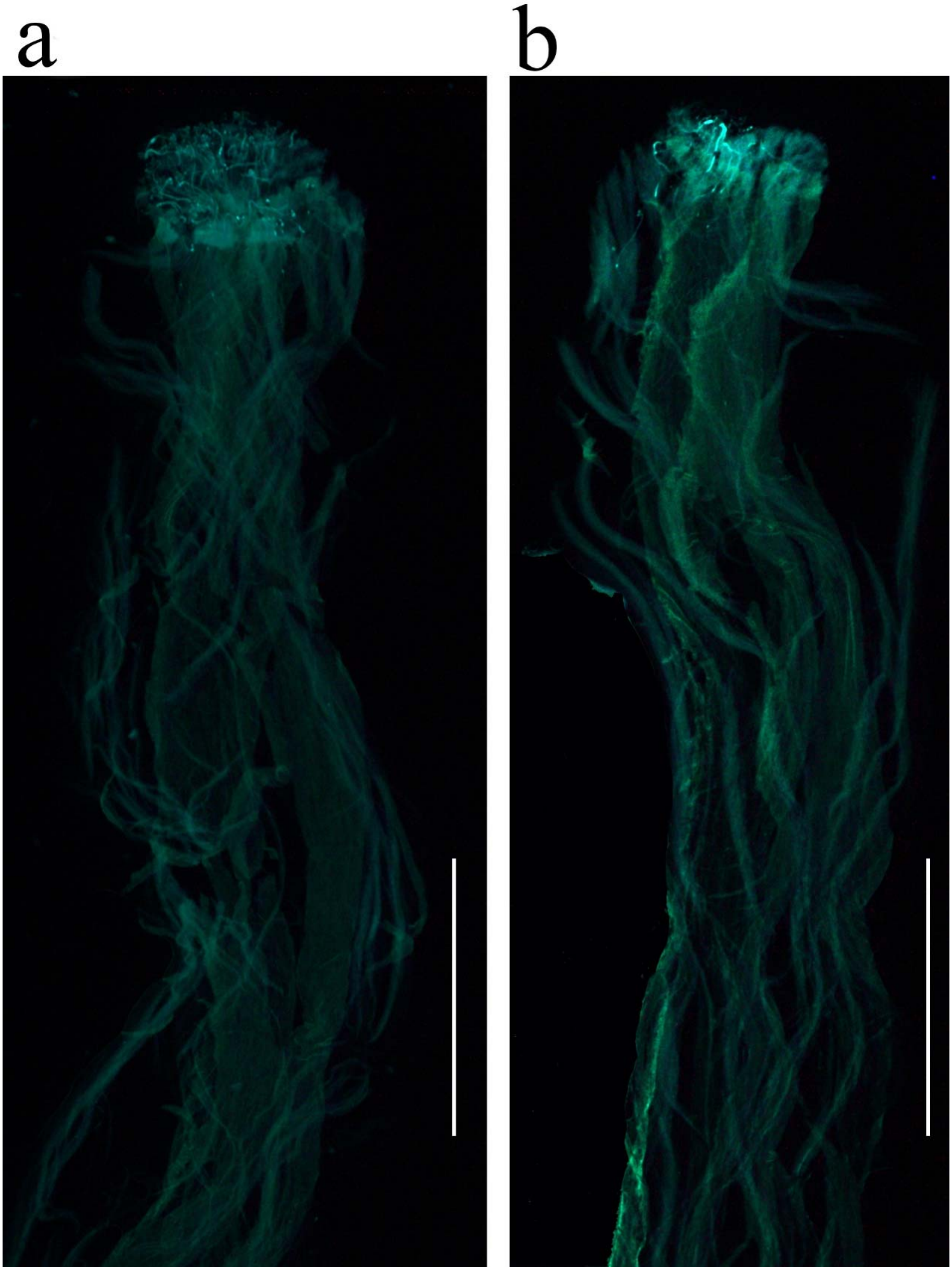

Supplement: Supplementary file 7 [file Image_3.pdf]
