# Supplementary material for: PLC-Mediated Signaling Pathway in Pollen Tubes Regulates the Gametophytic Self-incompatibility of Pyrus Species
Source: Front Plant Sci. 2017 Jul 6;8:1164. doi: 10.3389/fpls.2017.01164 (PMC5498517; doi:10.3389/fpls.2017.01164)
Supplement: Supplementary file 8 [file Image_4.pdf]

Supplementary figure S4. Statistics of pathway enrichment of differentially expressed proteins in each pairwise. RichFactor is the ratio of differentially expressed protein number annotated in this pathway term to all protein number annotated in this pathway term. Greater richFator means greater intensiveness. Pvalue ranges from 0~1, and less Pvalue means greater intensiveness. We just display the top 20 of enriched pathway terms.

Proteins usually interact with each other to play roles in certain biological functions. We perform pathway enrichment analysis of differentially expressed proteins based on KEGG database for each pairwise comparison. (a) Jinzhuili 0.5 h-Vs-Jinzhuili control; (b) Yali 0.5 h-Vs- Jinzhuili control.

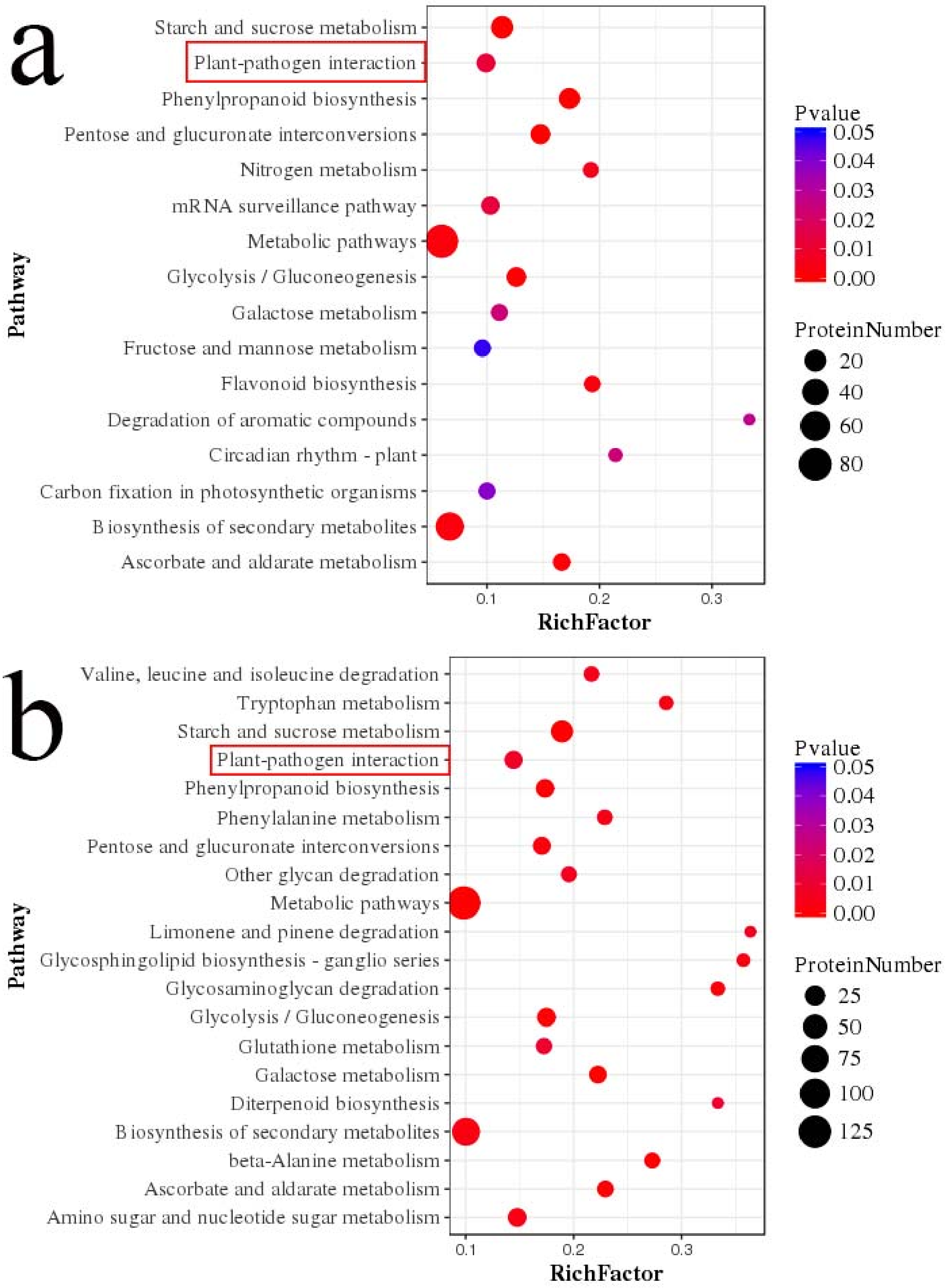
a
Starch and sucrose metabolism
Plant-pathogen interaction
Phenylpropanoid biosynthesis
Pentose and glucuronate interconversions
Nitrogen metabolism
mRNA surveillance pathway
Metabolic pathways
Glycolysis / Gluconeogenesis
Galactose metabolism
Fructose and mannose metabolism
Flavonoid biosynthesis
Degradation of aromatic compounds
Circadian rhythm - plant
Carbon fixation in photosynthetic organisms
Biosynthesis of secondary metabolites
Ascorbate and aldarate metabolism
Pathway
0.1
0.2
0.3
RichFactor
Pvalue
0.05
0.04
0.03
0.02
0.01
0.00
ProteinNumber
20
40
60
80
b
Valine, leucine and isoleucine degradation
Tryptophan metabolism
Starch and sucrose metabolism
Plant-pathogen interaction
Phenylpropanoid biosynthesis
Phenylalanine metabolism
Pentose and glucuronate interconversions
Other glycan degradation
Metabolic pathways
Limonene and pinene degradation
Glycosphingolipid biosynthesis - ganglio series
Glycosaminoglycan degradation
Glycolysis / Gluconeogenesis
Glutathione metabolism
Galactose metabolism
Diterpenoid biosynthesis
Biosynthesis of secondary metabolites
beta-Alanine metabolism
Ascorbate and aldarate metabolism
Amino sugar and nucleotide sugar metabolism
Pathway
0.1
0.2
0.3
RichFactor
Pvalue
0.05
0.04
0.03
0.02
0.01
0.00
ProteinNumber
25
50
75
100
125
